# Media Exposure and the Risk of Post-Traumatic Stress Disorder Following a Mass traumatic Event: An *In-silico* Experiment

**DOI:** 10.3389/fpsyt.2021.674263

**Published:** 2021-11-25

**Authors:** Salma M. Abdalla, Gregory H. Cohen, Shailesh Tamrakar, Shaffi Fazaludeen Koya, Sandro Galea

**Affiliations:** Department of Epidemiology, School of Public Health, Boston University, Boston, MA, United States

**Keywords:** PTSD—post-traumatic stress disorder, mental health, social media, mass shooting, mass traumatic events, media exposure

## Abstract

**Introduction:** Following mass traumatic events, greater exposure to traditional media like television (TV) about the event is associated with higher burden of post-traumatic stress disorder (PTSD). However, we know little about how social media exposure, combined with other media sources, shapes the population burden of PTSD following mass traumatic events.

**Materials and Methods:** We built a microsimulation of 1,18,000 agents that was demographically comparable to the population of Parkland and Coral Springs, Florida that experienced the Stoneman Douglas High School shooting in 2018. We parametrized the model using data from prior traumatic events and built an internal social network structure to facilitate the estimation of community PTSD prevalence following exposure to TV and social media coverage of the shooting.

**Results:** Overall, PTSD prevalence in the community due to exposure to TV coverage of the shooting was 3.1%. Shifting the whole population's hours of TV watching to the lower half of the population distribution decreased PTSD prevalence to 1.3% while increasing TV watching to the upper half of the distribution increased the prevalence to 3.5%. Casual (i.e., viewing posts) social media use in addition to exposure to TV coverage increased PTSD prevalence to 3.4%; overall prevalence increased to 5.3% when agents shared videos related to the shooting on social media.

**Conclusion:** This microsimulation shows that availability and exposure to media coverage of mass traumatic events, particularly as social media becomes more ubiquitous, has the potential to increase community PTSD prevalence following these events. Future research could fruitfully examine the mechanisms that might explain these associations and potential interventions that can mitigate the role of media in shaping the mental health of populations following traumatic events.

## Introduction

There is abundance of evidence indicating that mass traumatic events—such as natural disasters, large-scale attacks, and disease outbreaks—are associated with long lasting psychological consequences including depression and post-traumatic stress disorder (PTSD) (1–6). A recent review of the literature offers a wide range of PTSD prevalence estimates following a mass-shooting event ranging from 3% among parents of children exposed to a mass shooting to 91% among children in the same analysis (7). Exposure to traditional media coverage (including television (TV) and radio) of such events has also been associated with higher burden of mental disorders, both among persons who were directly affected by the event, and in the general population (8–11).

The emergence of social media as a ubiquitous presence introduces a new question: How does exposure to social media coverage of mass traumatic events affect the burden of PTSD in a community following a mass traumatic event?

Early evidence suggests that social media viewing of coverage of mass traumatic events is associated with adverse psychological consequences (12–14). However, the literature on the subject remains sparse. Given the increasing popularity of broadcasting trauma-related news on both 24-news cycles on TV and on social media, it is important to better understand how media exposure shapes the burden of PTSD in a population following mass traumatic events.

This is particularly relevant as the COVID-19 pandemic unfolds as a mass traumatic event with mental health consequences (2, 5, 15–19). For example, a recent study showed that persons who were exposed to COVID-19 related information on social media frequently had higher odds of anxiety compared to those with less exposure to COVID-19 related information on social media (20).

To help address this gap in our knowledge, we developed an *in-silico* experiment using a microsimulation. Public health often relies on linear methods to investigate the relationship between exposures and outcomes. Over the past century, however, there has been increasing appreciation of methods—such as agent-based modeling (ABMs) microsimulations—that aim to address the complexity of populations and the role of complexity in shaping population health. ABMs simulate the behaviors of autonomous “agents.” These agents represent individuals who then interact and form a synthetic population of interest, allowing for macro-level behaviors to emerge. They are appropriate for research questions that are based on a complex set of individual attributes, inter-agent interactions, and environment, as is the case with gun violence. Simulations allow for the development of counterfactual estimates, when given appropriate inputs, especially in the absence of real-world data on a subject (21). They have been used to answer many complex questions in public health including assessment of the impact of different policies on population health and health inequities; the role social networks and neighborhoods play in population levels of obesity; and potential interventions to reduce risky health behaviors at a population level (22). More recently, ABMs have been used in relation to mass traumatic events and population mental health (23, 24). In this paper we used a simulation to examine the role of exposure to TV and social media coverage of a mass traumatic event in shaping community PTSD prevalence.

## Materials and Methods

### Simulated Population

To ground our microsimulation in a recent mass traumatic event, we simulated the Stoneman Douglas High School (Parkland) shooting in 2018, which left 17 persons dead, 17 injured, and catalyzed a national conversation on the consequences of gun violence in the country, leading to widespread social media coverage. We initialized a population of 1,18,000 agents that was demographically comparable to the population of Parkland and Coral Springs, Florida, where the shootings took place. The demographic (sex, age, and race/ethnicity) parameters for this synthetic population were applied using data derived from the 2010–2015 American Community Survey (ACS) 5-year population estimates for the area (25).

### Level of Exposure

We designated within our model three levels of exposure to the event: primary, secondary, and tertiary. Primary exposure included agents who were injured or were present and in danger of being victims at the site of the mass shooting (900 students and 30 adults). We then built an internal social network to connect those directly affected with agents assigned as family or close friends. These 4,725 agents were assigned secondary level exposure in our simulation. Tertiary exposure included 1,12,189 adults living in the affected community at large but were directly affected or assigned as family or close friends to those directly affected. Using those in the tertiary exposure group, we simulated patterns of social media exposure, alone or coupled with TV exposure, and the frequency of such exposure during the first week following the mass shooting.

### Media Exposure

We used data from the Pew research center to calculate the percentage of agents to randomly assign TV as the preferred media outlet to receive news, stratified by age group (Supplementary Material).

Among agents who watched TV as their preferred news outlet, we used empirical data from a prior analysis to assign the number of hours of TV coverage of the traumatic event watched by agents (9). We also used data from another analysis to randomly assign PTSD status to agents based on their exposure to TV coverage of the event (11). To assess the added psychological association of exposure to social media among agents that watched TV, we used data from an analysis that examined the added association of sharing posts on social media and of viewing videos about the mass traumatic event (14).

### Shifting Population Distribution of Media Coverage

We implemented multiple scenarios that shifted the total number of hours of using TV in the population to estimate the potential association of changing media exposure on PTSD prevalence in the population. These included the following scenarios: in scenario 1 agents watched TV <4 h per day, in scenario 2 all agent preferences were shifted to the lower half of population TV watching distribution (i.e., all agents either watched 4 or fewer or 4–7 h of TV coverage), in scenario 3 all agent preferences were shifted to the upper half of population TV watching distribution (i.e., all agents either watched 8–11 or 12 or more h of TV coverage).

### Technical Details

The microsimulation was developed using C++ and implemented using Microsoft Visual Studio 2012 (Microsoft Corp). We ran each model scenario 50 times to account for stochasticity in the modeling process, with mean statistical measures reported. We then computed the 2.5th and 97.5th percentiles across those 50 simulations. The Supplementary Material provides an overview of the design concept and detailed protocol for this study including sub-models, pseudocodes, and a more in-depth focus on the microsimulation design concepts (Appendix 1).

## Results

Our simulated synthetic population closely resembled the 2010–2015 ACS 5-year age, sex, race/ethnicity, and education population distribution of Parkland and Coral Springs, Florida (Table 1). Overall, PTSD prevalence in the population based on exposure to TV coverage of the shooting was 3.1%.

**Table 1:** Comparison of Broward County American Community Survey (ACS) estimates with model synthetic population.

**Demographic group**	**Broward County, FL population (2010–2015 ACS Estimates) % (*N* = 1,843,152)**	**Model Population %(*N* = 1,728,265)**
**Sex**		
Female	51.452	51.142
Male	48.548	48.858
**Age**		
Under 20 years	24.01	24.38
20 to 44 years	33.15	33.38
45 to 64 years	27.83	27.74
65 and over	15.01	14.50
**Race/Ethnicity**		
White (Non-hispanic)	40.40	40.07
Hispanic	26.976	26.78
Black (Non-hispanic)	26.90	27.20
Asian (Non-hispanic)	3.43	3.45
American Indian and Alaskan Native (Non-hispanic)	0.17	0.24
Some other race (Non-hispanic)	0.45	0.52
Two or more race (Non-hispanic)	1.67	1.74
**Education**		
Up to 12th grade	12.36	12.64
High School	27.83	28.09
Some College	21.77	21.54
Associate's degree	9.62	9.64
Bachelor's degree	18.48	18.44
Graduate degree	9.94	9.65

### TV Coverage Interventions and PTSD Prevalence

**Figure 1:**
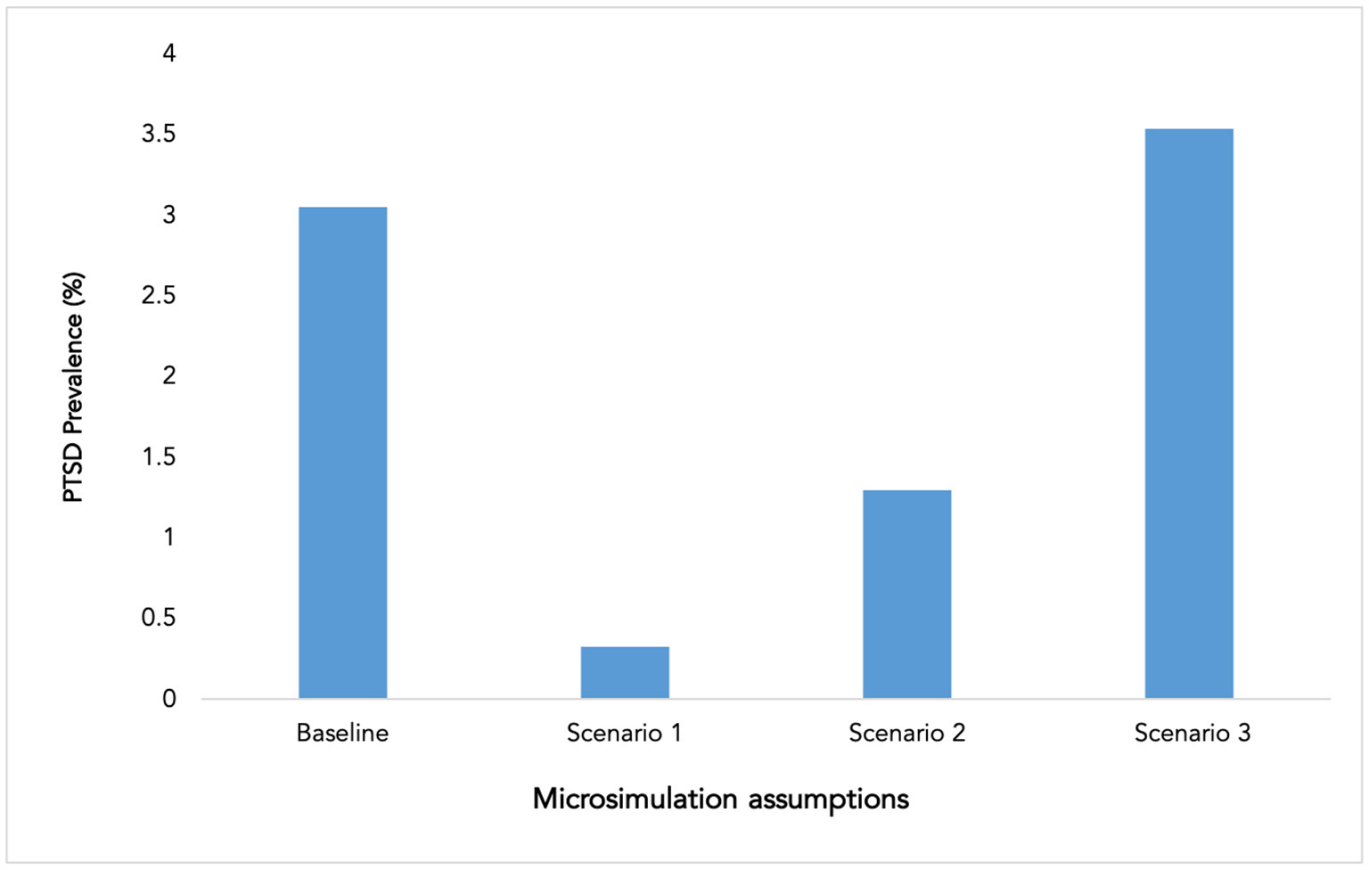
Exposure to television coverage of Parkland mass shooting and post-traumatic stress disorder (PTSD) prevalence. Baseline included a distribution of 11.2% agents watching <4 h, 13.1% watching between 4–7 h, 10.8% watching between 8–11 h, and 64.9% watching 12 h or more. Scenario 1: agents watched television <4 h per day. Scenario 2: all agent preferences were shifted to the lower half of population television watching distribution (i.e., all agents either watched 4 or less h or 4–7 h of television coverage). Scenario 3: all agent preferences were shifted to the upper half of population television watching distribution (i.e., all agents ether watched 8–11 h 12 or more hours of television coverage).

Compared to the baseline, moving the distribution of agent preferences for the number of hours watching TV to lower daily exposure (scenarios 1 and 2) was associated with a decrease in PTSD prevalence to 0.3 and 1.3% in scenarios 1 and 2, respectively. Shifting agent preferences to higher daily TV coverage exposure (scenario 3) was associated with an increase in PTSD prevalence to 3.5% (Figure 1).

### Social Media Coverage in Addition to TV Coverage

**Figure 2:**
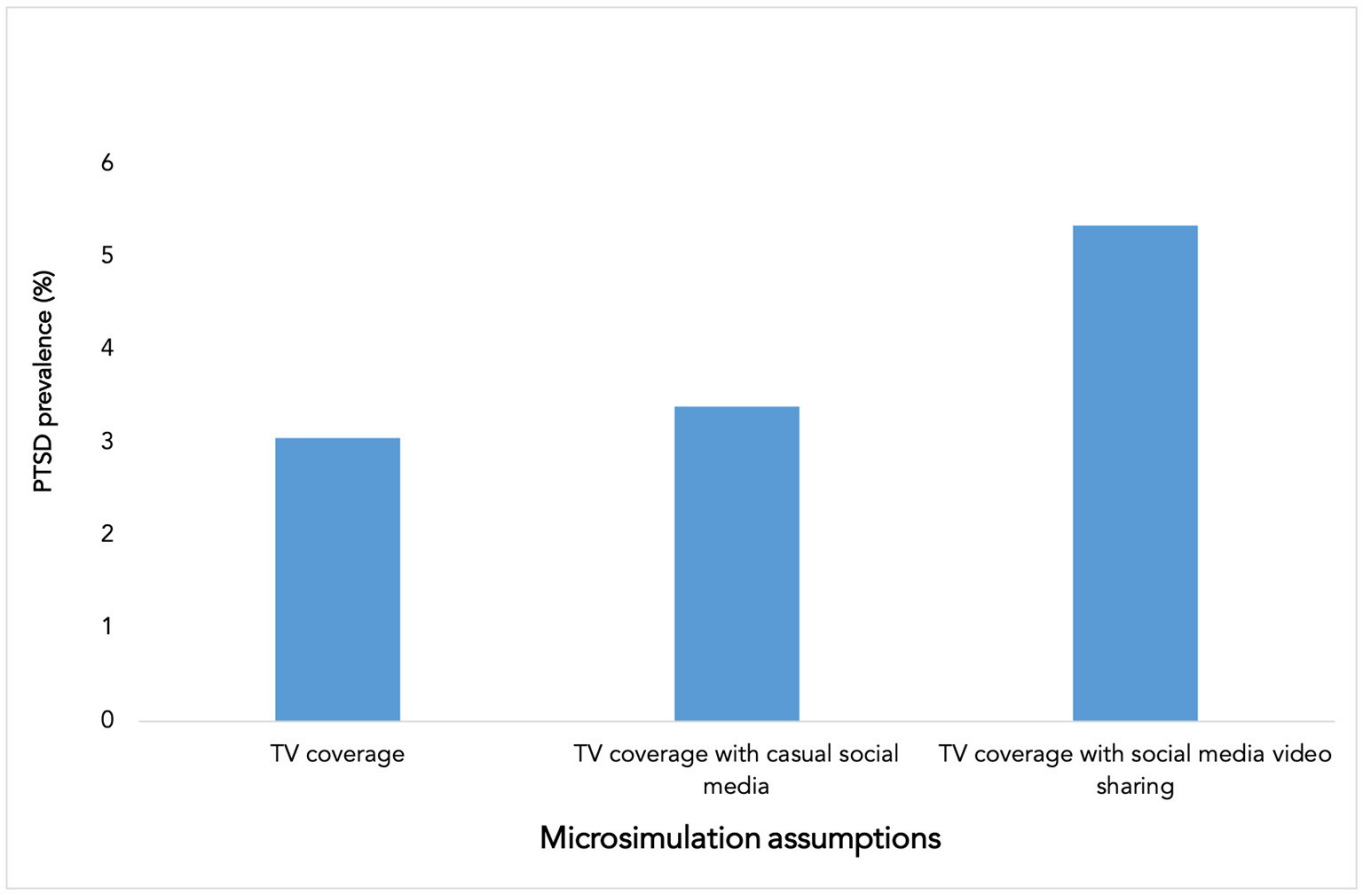
Exposure to television and social media coverage of the Parkland mass shooting and probable post-traumatic stress disorder (PTSD) prevalence. Casual social media use refers to viewing posts.

Adding casual social media use (scrolling through social media posts about the event) to watching TV led to an increase in the prevalence to 3.4%. When agents were assigned watching videos about the news on social media in addition to TV coverage, the prevalence of PTSD increased to 5.3% (Figure 2).

## Discussion

Using a microsimulation, we estimated community PTSD prevalence based on exposure to TV and social media content in Parkland and Coral Springs, Florida following the Parkland mass shooting of 2018. There were two key observations. First, decreasing population level TV exposure is associated with significant reductions in population PTSD prevalence and, second, exposure to social media coverage in addition to TV coverage can increase PTSD prevalence.

These findings suggest that decreasing TV exposure can be associated with a substantial reduction in population PTSD prevalence. Reducing TV coverage exposure in this simulation led to up to 90% decrease in prevalence in the general population. By contrast, increasing TV exposure was associated with a modest (15%) increase in PTSD prevalence. This can potentially be explained by the TV exposure distribution in the population, with the majority of the population (75.6%) already experiencing high exposure levels (8–11 and 12 and more h) as a baseline. This suggests that efforts to reduce TV exposure of mass traumatic events have the potential to substantially improve population mental health. These results build on other studies that show an increase in PTSD prevalence as the number of hours spent on watching TV coverage increases. For example, following the September 11 terrorist attack, an analysis showed the prevalence of PTSD increased as the number of hours spent on watching TV daily after the attack increased. PTSD prevalence among those who watched four h or less was 0.8% and the prevalence was as high as 10.1% among those who watched TV for 12 h or more (11).

We found that adding social media exposure to TV exposure can lead to a greater burden of PTSD, which almost doubles when the social media engagement includes sharing videos about the event. This echoes emerging evidence that exposure to social media relevant to traumatic events is associated with increased PTSD prevalence. For example, following the social unrest in Hong Kong in 2019, Ni et al. found that PTSD prevalence in the community was affected by the number of hours spent on relevant social media per day. PTSD prevalence was 7.2% among non-users, 12.6% among those who used social media for <2 h per day, and 23.5% among those who used social media to follow sociopolitical news for 2 h or more per day (26). Following Typhoon Hato in Macao, China, indirect exposure to information relevant to the typhoon through social media content about the typhoon or emotional reactions of those directly affected was associated with higher odds of PTSD (14). The increase in PTSD prevalence when assigning video watching compared to casually engaging with social media posts can potentially be explained because sharing videos may represent more investment in the particulars of the traumatic event. It is also possible that images are more evocative of a traumatic event experience and as such more salient as a traumatic stressor. This would be consistent with observations about the importance of particular visual images for PTSD in the aftermath of the September 11, 2001 terrorist attacks (9).

### Limitations

Our analysis should be considered with limitations in mind. First, and importantly, this was a simulation, with limited generalizability. The findings are based on the design and assumptions of our model, which are based on the results of several epidemiological studies. Each of these studies has its own limitations. However, the peer-reviewed studies we used to provide parameters afford confidence in the estimates provided by our simulation. We also aimed to stratify media exposure by age group within the population to provide a more accurate distribution of PTSD prevalence that accounts for the differences in media outlet preferences in the population. We see this simulation as essential, formative work, paving the way for more empirical research on the topic. Second, these results only apply to those who use TV as their preferred source of news. Given the absence of empiric data, we could not account for the PTSD prevalence among those who receive their news from other sources. Third, while our analysis aims to differentiate between two levels of social media engagement, we do not offer insight about how different types of exposure (e.g., news updates vs. photos of victims) could affect PTSD prevalence. This is due to the paucity of empirical data around the subject, limiting our ability to add these assumptions to the model.

### Conclusion

Using a microsimulation, we document the relative contribution of exposure to TV and social media coverage of a mass shooting to community PTSD prevalence. We found that the constant availability and exposure to media coverage of mass traumatic events through either 24-h news cycle TV and social media, particularly as social media becomes more ubiquitous, has the potential to increase the burden of community PTSD following such events. We note that while TV remains the main source for news in the US, use of social media has been on rise over the past decade, suggesting that our findings would change as news consumptions preferences change, particularly if social media becomes a dominant source of news consumption coupled with TV over time (27). To that end, future research should focus on further quantifying the role of social media, and other media outlets, in shaping PTSD prevalence following mass traumatic events and the potential interventions that could mitigate the role media plays in shaping the mental health consequences of mass traumatic events.

## Data Availability Statement

The original contributions presented in the study are included in the article/Supplementary Material, further inquiries can be directed to the corresponding author.

## Author Contributions

SA and GC designed the research and gathered data. ST conducted the data analyses with support from SA and GC. SA, GC, and SFK interpreted the data with support from SG. SA wrote the first draft of the manuscript. SG read and revised the first draft. All authors read and approved the final manuscript.

## Conflict of Interest

The authors declare that the research was conducted in the absence of any commercial or financial relationships that could be construed as a potential conflict of interest.

## Publisher's Note

All claims expressed in this article are solely those of the authors and do not necessarily represent those of their affiliated organizations, or those of the publisher, the editors and the reviewers. Any product that may be evaluated in this article, or claim that may be made by its manufacturer, is not guaranteed or endorsed by the publisher.
