# Supplementary material for: Media Exposure and the Risk of Post-Traumatic Stress Disorder Following a Mass traumatic Event: An In-silico Experiment
Source: Front Psychiatry. 2021 Nov 25;12:674263. doi: 10.3389/fpsyt.2021.674263 (PMC8656276; doi:10.3389/fpsyt.2021.674263)
Supplement: Supplementary file 1 [file Data_Sheet_1.PDF]

eAppendix: Description of the model

1 Purpose

The purpose of this eAppendix is to provide a comprehensive detail on the implementation of the microsimulation model that assesses the affects of media coverage of mass-shooting on incidence of PTSD among community members.

2 Entities, state variables and scales

Each person in the model has a unique set of static and time-varying state variables. The variables age, race, and gender define its demographic attributes. They are also assigned variables related to PTSD (type and case status) and media exposure (such as news source and time spent on watching the news).

3 Initialization

The initialization steps are described in detail below:

3.1 Create synthetic population

We created an artificial population of Parkland and Coral Springs, FL by using an approach called IPU (Iterative Proportional Updating) developed by a group in Arizona State University. This method computes the selection probabilities of different household types (cross-tabulation of households based on household type, size and income) by iteratively adjusting weights until both household-level and person-level estimates match joint distributions obtained from IPF (Iterative Proportion Fitting) procedure. The details about this method is described elsewhere [1]. Agent attributes (age, race, gender and education) are assigned based on selection probabilities computed with IPU.

3.2 Create school

We randomly picked agents from our synthetic population to build our virtual school based on demographics of Marjory Stoneman Douglas High School obtained from National Center of Education Statistics.

3.3 Create social network

In this step, social network of agents is created by matching their attributes based on age difference, race, gender and educational attainment. For example, two agents belonging to same age group and attending same school are more likely to be friends with each other.

3.4 Group agents by exposure level

In this step, we create a pool of agents based on the exposure to the shooting. Agents exposed directly to the shooting were classified under primary exposure. Family and close friends of directly affected agents were classified under secondary exposure and, finally tertiary exposure included community members who were indirectly affected by shooting.

3.5 Distribute the news source

The source of news on mass-shooting were assigned to community members (agents) based on age-specific distribution as shown the table below:

Table 1: Distribution of the news source

Age range	TV only	Social Media only	TV and Social Media	Others
18 - 29	0.145	0.290	0.011	0.554
30 - 49	0.272	0.230	0.026	0.472
50 - 64	0.588	0.145	0.044	0.223
65+	0.791	0.106	0.028	0.075

A random number generated between 0 and 1 is compared against the distribution provided in the table above. For example, if random number is less than 0.145 for an agent belonging to 18-29 age group, TV is assigned as a primary source of news for that agent.

3.6 Distribute hours of TV coverage

Community members (agents) with primary source of the news as TV are also assigned the time they spend on TV news coverage related to the shooting. The distribution of population consuming the news coverage of the shooting by the hour is provided in the table below:

Table 2: Distribution of TV coverage

TV Coverage (Hours)	Proportion
≤ 4	0.1125
4 -7	0.1312
8 - 11	0.1078
≥ 12	0.6486

For example, if a random number generated between 0 and 1 is less than 0.1125, agent spent less than 4 hours watching the news on mass-shooting.

3.7 Distribute PTSD status

PTSD status acts as a marker that distinguishes positive cases from negative cases. Community members with TV as a source of news are assigned PTSD status. The table below shows the probability of developing a PTSD based on hours of TV news coverage of the shooting for the agents who - 1) Watch TV only, 2) Watch TV and scroll social media posts and 3) Watch TV and share video in social media.

Table 3: Prevalence of PTSD by hours of TV coverage

TV Coverage (Hours)	Probable PTSD (TV only)	Probable PTSD (TV + Social Media Scrolling), OR = 1.11	Probable PTSD(TV + Video Sharing), OR = 1.75
≤ 4	0.008	0.0088	0.014
4 -7	0.039	0.04329	0.06825
8 - 11	0.042	0.04662	0.0735
≥ 12	0.101	0.1121	0.17675

4 Input data

The model did not require input data beyond what was provided in initialization, since the environment remained constant throughout the simulation.

5 Scenarios

We ran a series of hypothetical scenarios by adjusting hours of TV news coverage and assessed the impact it had on the prevalence of age-specific PTSD. The list of scenarios are described in detail below:

5.1 Scenario 1

In this scenario, we changed the distribution of TV news coverage as shown in the table below so that all the agents had less than four hours of TV news coverage.

Table 4: Distribution of TV coverage

**TV Coverage (Hours)**	**Proportion**
≤ 4	1.00
4 -7	0.00
8 - 11	0.00
≥ 12	0.00

5.2 Scenario 2

In this scenario, the hours were shifted to higher half of the distribution as shown in the table below.

Table 5: Distribution of TV coverage

**TV Coverage (Hours)**	**Proportion**
≤ 4	0.000
4 -7	0.000
8 - 11	0.2203
≥ 12	0.7798

5.3 Scenario 3

In this scenario, the hours were shifted to lower half of the distribution as shown in the table below.

Table 6: Distribution of TV coverage

**TV Coverage (Hours)**	**Proportion**
≤ 4	0.2203
4 -7	0.7798
8 - 11	0.0000
≥ 12	0.0000

6 Summary of agent characteristics and initialization parameters

1. Agent characteristics, values and description

Agent characteristics	Values	Description
Age	Age1 (14-17)<br>Age2 (18-34)<br>Age3 (35-64)<br>Age4 (65+)	
Gender	Male<br>Female	
Race	White non-Hispanic<br>Black non-Hispanic<br>Other non-Hispanic<br>Hispanic	
Number of friends	Variable	Assigned with Poisson distribution with mean value of 3
Exposure level	Primary<br>Secondary<br>Tertiary	
PTSD status	Positive<br>Negative	
News Source	TV<br>Social Media<br>TV and Social Media<br>Others	
Hours of TV coverage	≤ 4<br>4 - 7<br>8 - 11<br>≥ 12	

7 Model flow charts

1. Flow chart indicating steps in model initialization

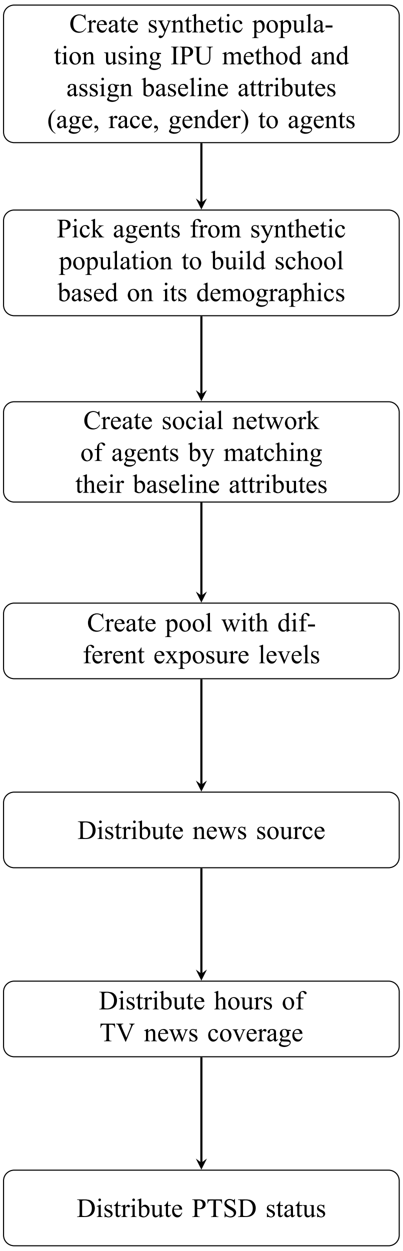

8 Pseudo-code

1. Creating synthetic population

Algorithm 1 Synthetic population

```
procedure createPopulation()
    compute selection probabilities for households (by type, size and income) using IPU
    Import household-level PUMS dataset
    for household type in PUMS households list do
        num_household = household count by household type
        while (num_household >0)  do
            random = rand(0, 1)
            for id, probability in household type do
                if (random <probability) then
                    household = households[id]
                    for person in household do
                        add person to list
                    end for
                    break
                end if
            end for
        end while
    end for
```

2. Creating school and community

Algorithm 2 School demographics

```
procedure createSchool()
    Import school demographics
    for household in synthetic households list do
        for person in household do
            if Student then
                student_count = count from school demographics by student type
                if student_count >0 then
                    add person to school as a student
                    student_count - -
                end if
            if Teacher then
                teacher_count = count of teachers in school
                if teacher_count >0 then
                    add person to school as a teacher
                    teacher_count - -
                end if
            if Others then
                add person to community list
            end if
        end for
    end for
```

3. Creating social network

Algorithm 3 Social network

```
procedure socialNetwork()
    for i = agent list start to agent list end do
        Agent a = assign agent i to agent a
        max_friends = assign maximum friends agent a can have
        while (agent a reaches max_friends) do
            Agent b = randomly pick agent i from the list
            if (a is friend of b OR a and b are family) then
                do nothing
            else
                Age_Match = False; Gender_Match = False
                Race_Match = False; Education_Match = False
            if (random(0,1) >0.185) then //Age check
                if (age difference between a and b <= 10) then
                    Age_Match = True
            if (random(0,1) >0.995) then // Gender check
                if (agent a and agent b are of same sex) then
                    Gender_Match = True
            if (random(0,1) >0.01) then // Race check
                if (agent a and agent b are of same race) then
                    Race_Match = True
            if (random(0,1) >0.25) then //Education check
                if (agent a and agent b have same education level) then
                    Education_Match = True
            if (Age_Match & Gender_Match & Race_Match & Education_Match) then
                add agent b to agent a friend's list
                add agent a to agent b friend's list
            end if
        end while
    end for
```

4. Assignment of the news source

Algorithm 4 News Source

```
procedure setNewsSource()
    Import news source distribution by age-category
    for person in community members do
        random = rand(0, 1)
        if random <probability TV news  then
            newsSource = TV
        end if
        if random <probability social media then
            newsSource = Social Media
        end if
        if random <probability TV and social media then
            newsSource = TV and social media
        end If
        if random <probability others then
            newsSource = Others
        end If
    end for
```

5. Assignment of TV news coverage hours

Algorithm 5 TV coverage hours

```
procedure setTvHours()
    Import TV coverage hours distribution
    for person in TV watchers do
        random = rand(0, 1)
        if random <probability (<4 hrs)  then
            tvHours = assign <4 hrs
        end if
        if random <probability (4 - 7 hrs) then
            tvHours = assign 4 - 7 hrs
        end if
        if random <probability (8 - 11 hrs) then
            tvHours = assign 8 - 11 hrs
        end if
        if random <probability (>12 hrs) then
            tvHours = assign >12 hrs
        end if
    end for
```

6. Assignment of PTSD status

Algorithm 6 PTSD status

```
procedure setPtsdStatus()
    Import PTSD prevalence by hours of TV news coverage
    for person in TV watchers do
        TV hours = Time spent by person p on TV news
        random = rand(0, 1)
        if random <ptsd_prevalence[TV hours] then
            PTSD status = POSITIVE
        else
            PTSD status = NEGATIVE
        end If
    end for
```

9 Results

9.1 Age-specific PTSD prevalence at baseline for TV only

Age group	Prevalence(%)
18 - 29	1.1106
30 - 49	2.0757
50 - 64	4.4527
65+	6.0075

9.2 Age-specific PTSD prevalence for TV only - Scenario 1: Shifting media coverage distribution to <4 hours

Age group	Prevalence(%)
18 - 29	0.1174
30 - 49	0.2202
50 - 64	0.4677
65+	0.6270

9.3 Age-specific PTSD prevalence for TV only - Scenario 2: Shifting media coverage distribution to upper half

Age group	Prevalence(%)
18 - 29	1.2774
30 - 49	2.3990
50 - 64	5.1663
65+	6.9405

9.4 Age-specific PTSD prevalence for TV only - Scenario 3: Shifting media coverage distribution to lower half

Age group	Prevalence(%)
18 - 29	0.4683
30 - 49	0.8829
50 - 64	1.8927
65+	2.5057

References

[1] X. Ye, K. Konduri, R. M. Pendyala, B. Sana, and P. Waddell, "A METHODOLOGY TO MATCH DISTRIBUTIONS OF BOTH HOUSEHOLD AND PERSON ATTRIBUTES IN THE GENERATION OF SYNTHETIC POPULATIONS," tech. rep.
